# Evaluating diverse electronic consultation programs with a common framework

**DOI:** 10.1186/s12913-018-3626-4

**Published:** 2018-10-24

**Authors:** Delphine S. Tuot, Clare Liddy, Varsha G. Vimalananda, Jennifer Pecina, Elizabeth J. Murphy, Erin Keely, Steven R. Simon, Frederick North, Jay D. Orlander, Alice Hm Chen

**Affiliations:** 1 Center for Innovation in Access and Quality at Priscilla Chan and Mark Zuckerberg San Francisco General Hospital and Trauma Center, University of California San Francisco, San Francisco, CA 94110 USA; 2 C.T. Lamont Primary Health Care Research Centre, Bruyère Research Institute, Ottawa, ON Canada; 3 Department of Family Medicine, University of Ottawa, Ottawa, ON Canada; 4 Center for Healthcare Organization and Implementation Research, Edith Nourse Rogers Memorial VA Medical Center, Bedford, MA USA; 5 Department of Medicine, Boston University School of Medicine, Boston, MA USA; 6 Department of Family Medicine, Mayo Clinic, Rochester, MN USA; 7 Department of Medicine, University of Ottawa, Ottawa, ON Canada; 8 The Ottawa Hospital, Ottawa, ON Canada; 9 Harvard Medical School, Boston, USA; 10 Department of Primary Care Internal Medicine, Mayo Clinic, Rochester, MN USA; 11 VA Boston Healthcare System, Boston, USA; 12 Deparment of Medicine, University of California, San Francisco, San Francisco, CA USA

**Keywords:** E-consult, Electronic consultation, Evaluation, Quadruple aim, RE-AIM

## Abstract

**Background:**

Electronic consultation is an emerging mode of specialty care delivery that allows primary care providers and their patients to obtain specialist expertise without an in-person visit. While studies of individual programs have demonstrated benefits related to timely access to specialty care, electronic consultation programs have not achieved widespread use in the United States. The lack of common evaluation metrics across health systems and concerns related to the generalizability of existing evaluation efforts may be hampering further growth. We sought to identify gaps in knowledge related to the implementation of electronic consultation programs and develop a set of shared evaluation measures to promote further diffusion.

**Methods:**

Using a case study approach, we apply the Reach, Effectiveness, Adoption, Implementation and Maintenance (RE-AIM) and the Quadruple Aim frameworks of evaluation to examine electronic consultation implementation across diverse delivery systems. Data are from 4 early adopter healthcare delivery systems (San Francisco Health Network, Mayo Clinic, Veterans Administration, Champlain Local Health Integration Network) that represent varied organizational structures, care for different patient populations, and have well-established multi-specialty electronic consultation programs. Data sources include published and unpublished quantitative data from each electronic consultation database and qualitative data from systems’ end-users.

**Results:**

Organizational drivers of electronic consultation implementation were similar across the systems (challenges with timely and/or efficient access to specialty care), though unique system-level facilitators and barriers influenced reach, adoption and design. Effectiveness of implementation was consistent, with improved patient access to timely, perceived high-quality specialty expertise with few negative consequences, garnering high satisfaction among end-users. Data about patient-specific clinical outcomes are lacking, as are policies that provide guidance on the legal implications of electronic consultation and ideal remuneration strategies.

**Conclusion:**

A core set of effectiveness and implementation metrics rooted in the Quadruple Aim may promote data-driven improvements and further diffusion of successful electronic consultation programs.

**Electronic supplementary material:**

The online version of this article (10.1186/s12913-018-3626-4) contains supplementary material, which is available to authorized users.

## Background

The demand for specialty care is growing and is predicted to exceed specialist workforce capacity in the United States by 2025 [1]. Electronic consultation, which provides a way for primary care providers (PCPs) and their patients to obtain specialty expertise without an in-person patient visit to the specialist, has emerged as a mode of specialty care delivery that may address the impending supply-demand mismatch for specialty care.

Studies of individual early adopter electronic consultation programs in North America have demonstrated benefits consistent with the overarching goal of achieving the quadruple aim [2]: high-quality care delivery that improves population health [3–5] and achieves high satisfaction among providers [6–8], care-team members, and patients, [9] while introducing cost-savings from new efficiencies [10, 11]. Two systematic reviews that included international programs have also reported that electronic consultation programs are associated with improved access to specialty care and enhanced care coordination, high satisfaction among primary and specialty care providers, and positive patient experience [12, 13].

Despite these data, electronic consultation programs have not achieved widespread use in the United States. The use of variable evaluation metrics across health systems and concerns related to the generalizability (or lack thereof) existing evaluation efforts may be hampering further growth, alongside financial and cultural barriers. Using a case study approach with four very different health delivery systems, we apply the Reach, Effectiveness, Adoption, Implementation and Maintenance (RE-AIM) [14] and the Quadruple Aim [2] frameworks of evaluation to highlight the generalizability of electronic consultation program implementation. In so doing, we also identify gaps in knowledge to promote areas of future study and propose a set of shared evaluation measures to promote future study.

## Methods

### Definitions

Electronic consultations (e-consults) are asynchronous provider-provider consultations that occur within an electronic health record (EHR) or a web-based portal. E-consults allow providers, most commonly PCPs, to access specialist input for clinical questions that may be addressable without an in-person specialty care visit. Electronic consultation systems allow specialists to address consultative needs through chart review and communication with the referring provider, and ensure adequate diagnostic workup prior to a face-to-face patient visit when necessary. There are three main types of electronic consultation systems. (1) They can exist independently or (2) in parallel to referral processes (either electronic – known as e-referrals -- or paper/fax based), which carry the expectation of a specialty care visit. (3) Alternatively, some are integrated into the overall referral process, creating integrated electronic consultation and referral systems, in which all requests for specialty expertise are electronically initiated and specialist reviewers respond to each request with the appropriate form of consultation, including scheduling the patient for an in-person visit.

### Study design and data sources

We used a case study design to explore the impact of electronic consultation system implementation across 4 diverse healthcare delivery systems. The systems were selected because they represent varied organizational structures, care for different patient populations, have well-established multi-specialty electronic consultation programs that represent the three types of systems, and have undergone independent evaluations of program implementation and local impact (Table 1). Data sources from each health delivery system included published quantitative data from each system’s electronic consultation database and qualitative data from each system’s end-users. Additionally, some unpublished data from each system were provided by co-authors.

**Table 1:** Characteristics of four health systems

Name	Geographic area	Type of organization	Patient population served annually	Type of electronic consultation and/or referral system	Year implemented	Technology platform
Champlain BASE eConsult service	Eastern Ontario	Community with non-affiliated PCP and specialist practices	1.3 million	independent electronic consultation system	2009 proof of concept; 2011 spread	Microsoft Sharepoint collaboration space on private web network
Mayo Clinic	Campuses in Rochester, MN; Scottsdale AZ; and Jacksonville, FL	Integrated academic medical center	1.3 million	parallel electronic consultation and referral systems	2008	embedded into enterprise-wide EMR
San Francisco Health Network	San Francisco, California	urban safety-net with affiliated PCPs and specialists	100,000	integrated electronic consultation and referral system	2005 pilot; 2007 spread	software application that is embedded with the hospital EMR but not all ambulatory EMRs
Veterans Administration	United States	Integrated public system	6 million	parallel electronic consultation and referral systems	2011	embedded into enterprise-wide EMR

### Evaluation frameworks: RE-AIM and quadruple AIM

The five constructs of the RE-AIM framework [14] (*Reach, Effectiveness, Adoption, Implementation, Maintenance*) formed the basis for analysis in this study along with the Quadruple Aim framework to further refine *Effectiveness* measures. RE-AIM is well-suited to our case study as it addresses critical aspects of implementation in real-world settings and identifies facilitating and hindering factors to success, which are particularly important for external validity. Additional file 1: Table S1 summarizes the dimensions of these validated frameworks with associated definitions and evaluation questions pertinent to electronic consultation programs.

The *Reach* and *Effectiveness* constructs reflect patient-level measures and together, they measure the impact of an intervention on a given population. *Reach* considers the extent to which a sample of participants reflects the overall eligible population for that intervention. *Effectiveness* measures reflect short-term and/or long-term patient-level outcomes, including unintended positive and negative consequences. They can be categorized into 4 domains using the Quadruple Aim framework: *Population Health, Patient Satisfaction*, *Care Team Satisfaction* and *Financial Implications* [2]. Measures of *Adoption* and *Implementation* are organizational measures that focus on translatability of an intervention to other settings. *Adoption* is related to the absolute number, proportion and representativeness of providers who use a new program. *Implementation* refers to the extent and fidelity of the intervention compared to intended design/workflow, including barriers and enablers to local implementation and program modifications for success. The *Maintenance* dimension of the framework addresses the extent to which the intervention is sustainably embedded within routine organizational practice (Additional file 1: Table S1).

## Results

### Case studies

Four case studies were performed, examining electronic consultation implementation in systems that represent different structures of specialty care delivery: a community setting with non-affiliated individual PCP and specialty care practices; an integrated tertiary care academic medical center; an urban safety-net system with affiliated PCPs and specialists; and a fully integrated public delivery system (Table 1).

#### Champlain local health integration network (LIHN)

The Champlain BASE™ (Building Access to Specialists through eConsultation) program was developed in 2009. It is based in the Champlain health region of Eastern Ontario, Canada, which has a population of 1.3 million individuals, roughly half of whom reside in the city of Ottawa [15]. Independent PCPs who sign up for the BASE service can submit e-consults to any specialist who has agreed to provide this service, from any internet-connected device via the service’s secure SharePoint platform, attaching any files they deem pertinent (e.g. images, test results). A BASE program manager allocates the e-consult to a specialist in the specialty group selected by the PCP. That specialist responds with expert advice, a recommendation for referral, or a request for additional information. This service exists in parallel to existing referral practices in the Champlain health region and is not embedded into any particular EHR. Specialist reviewers are remunerated for their e-consult activities using the fee schedule for in-person consultation pro-rated to time spent, from a combination of regional, provincial, and research funding.

#### Mayo Clinic

This is an academic not-for-profit tertiary care academic medical center with three major campuses in Rochester, Minnesota; Scottsdale, Arizona; and Jacksonville, Florida. E-consults for the primary care population were offered to PCPs in 2008. In 2010 the program was expanded to allow Mayo specialists to request e-consults from other Mayo specialists. The electronic consultation program exists in parallel to the electronic referral system that providers use to order face-to-face specialty visits; both systems are embedded into the EHR, so that specialist reviewers can review any background data to complete e-consults. Completed e-consults are available in the EHR and the patient portal for subsequent review by ordering providers and patients, respectively [15]. Specialist reviewers at the Mayo clinic are salaried, do not receive additional compensation to perform e-consults but do receive some protected time.

#### San Francisco Health Network (SFHN)

The SFHN is San Francisco’s publicly funded safety-net healthcare delivery system. All specialty services are provided at the acute care hospital, Zuckerberg San Francisco General Hospital and Trauma Center (ZSFG). Requests for specialty care come from fourteen SFHN primary care clinics and an affiliated network of twelve community health centers. The referral base consists of approximately 100,000 patients. Since 2007, all requests for ZSFG specialty services are initiated as e-consults through the electronic referral and consultation program that is embedded into the EHR [16]. ZSFG specialists are all salaried; specialist reviewers receive work credit for their e-consult activities.

#### Veterans administration (VA)

The VA is the largest integrated healthcare system in the United States, caring for over 6 million Veterans annually. Specialists are based at approximately 170 VA Medical Centers (VAMC). Most VAMCs are affiliated with one or more of the 1000 community-based outpatient centers located between a few to 300 miles from the parent facility. The VA launched its electronic consultation program in 2011 with variable implementation across the VAMCs. Among participating VAMCs, any provider with ordering privileges can request an e-consult within the EHR in the same manner as they would a traditional face-to-face referral; the two systems exist in parallel. Patient data is available for review within the EHR. Specialists have the option to convert e-consult requests to face-to-face referrals and vice versa. VA providers are usually salaried and specialists receive workload credit based on the time spent to complete each e-consult.

### Evaluation

Application of the RE-AIM and Quadruple Aim frameworks to the evaluation of each electronic consultation implementation is detailed below. Key data from each system pertinent to measures within each RE-AIM dimension and Quadruple Aim domain are also summarized in Table 2.

**Table 2:** Evaluation data pertinent to the Reach Effectiveness Adoption Implementation Maintenance (RE-AIM) framework for each delivery system

RE-AIM dimensions and Quadruple Aim domains and example measures	Champlain BASE	Mayo	San Francisco Health Network	Veterans Administration
REACH				
Approximate annual number of e-consults	10,000	18,000	46,500	443,600
Percentage of requests for specialty care among participating services, initiated an as e-consult	unknown	unknown	100%	2%
Approximate number of e-consults per 1000 patient-lives	8	14	465	74
Demographic information of patients who received an e-consult	84% adult, 16% pediatric	unknown	93% adult; 54% female; 29% Hispanic, 25% Asian, 20% White, 17% Black	32% were for patients older than 65 years; 55% female
EFFECTIVENESS				
Quadruple aim: Population Health				
E-consult response time	Mean response 1 day	Mean response 2 days	91% response within 3 days	95% within 3 days
Third next available in-person new patient appointment	unknown	unknown	Decreased after e-consult implementation	unknown
E-consult management: % e-consults without a face-to-face visit in the same specialty	71%	82%	23%	37%
Quality of care: specialty-specific patient-level outcomes	unknown	unknown	unknown	unknown
Quality of care: provider perceptions	92% PCPs believed that overall value of e-consults to patients was excellent/very good; 56% of specialists believed that e-consults improved access to care	73% of PCPs agree e-consults provide “good medical care”	72% PCPs agree/strongly agree that e-consults improve clinical care	56% PCPs obtained specialty input for patients who would not travel to see a specialist; 61% specialists agree that e-consult provide “high quality medical care”
Educational value for referring providers	93% of PCPs report high educational value	unknown	84% of PCPs report that e-consults have educational value	unknown
Quality of care: potential harms/safety-implications	3.4% of e-consult cases led to initiation of a face-to-face referral when one was not originally considered	10% of specialty recommendations were not completed by PCPs	1–2% of patients who received a gastroenterology or general surgery e-consult experienced unintended emergency department visits or hospital admissions	6.3% e-consults lacked appropriate specialist follow-up after initial communication; 7.4% of PCPs did not appropriately follow-up
Quadruple Aim: Patient experience	46% considered e-consult a viable alternative to an Endocrine face-to-face visit	unknown	Patients identified benefits to e-consults and a desire for more information about the PCP-specialist communication	Median satisfaction score of 5 on a 5-point Likert scale
Quadruple Aim: Care team experience	95% of PCPs reported high satisfaction; Interview data suggest high PCP and specialist satisfaction	80% of PCPs reported good or excellent satisfaction with e-consults	80% of PCPs agree/strongly agree that they are satisfied; qualitative data from specialists suggest high satisfaction	93% of PCPs and 53% of specialists are satisfied; qualitative data suggest high satisfaction
Quadruple Aim: Financial implications	Cost savings from decreased specialty care visits	unknown	unknown	Decreased costs related to patient travel
ADOPTION				
Number of e-consult specialty services	92	53	55	Over 50, varies by region
Types of e-consult specialty services	Medical, Surgical, Women’s health, Pediatric, Mental Health	Medical, Surgical, Women’s health, Pediatric	Medical, Surgical, Women’s health, Pediatric	Medical, Surgical, Women’s Health, Mental Health
Number and percentage of PCPs using the service	75% (*n* = 1240)	96% (*n* = 350)^a^	100% (*n* = 76)^b^	unknown
Characteristics of PCPs using the service	Family physicians, Internal Medicine physicians (in the U.S.), Nurse Practitioners, Physician Assistants, General pediatricians (Mayo, SFHN)			
IMPLEMENTATION				
Predisposing drivers for implementation	Supply-demand mismatch for specialty care with resulting poor access to specialty services	Desire to improve access for in-person specialty care visits and expand primary care scope to manage more complex patients	Supply-demand mismatch for specialty care with resulting poor access to specialty services; inefficient referral process	Variable access to specialty services
Reinforcing organizational factors	Identification of specialty champions	Integration in to EHR; automated e-consults for certain clinical situations	Primary care workflow re-design; inclusion of trainees in the e-consult workflow; mandatory for all requests for specialty care	Primary and specialty care workflow re-design; identification of specialty champions; local autonomy to develop new templates and workflows
Barriers to implementation	Legal implications; lack of clinical oversight	Increased specialist workload; changes in specialist workflow; variation in how specialties value the work involved	Increased primary care workload and changes in workflow; lack of clinical oversight; legal implications	Increased PCP and specialist workload; lack of widespread training
MAINTENANCE				
Inclusion into routine practice	yes	yes	yes	yes
Reinforcing individual-level factors	Remuneration of PCPs and specialists per e-consult	Salaried specialists who receive work credit	Salaried specialists who receive work credit	Salaried specialists who receive work credit
Reinforcing system-level factors	Dedicated project team for customer service; ongoing quality improvement; regional healthcare policy buy-in	Ongoing quality improvement	Dedicated project team for onboarding, dissemination, and analysis; executive leadership	Local autonomy to develop new workflows; executive leadership; strong direct communication and pre-existing relationships between PCPs and specialists

^a^Data are pertinent to the Rochester site only

^b^Data are pertinent to SFHN primary care clinics only

#### Reach

Across the four systems, the annual number of e-consults ranges from 10,000 to 443,600, representing a range of e-consults/1000-patient lives of 1 to 378. Demographic characteristics of patients who receive e-consults have been sparsely published, though VA data suggest that patients who received an e-consult lived in more rural areas than those who received a face-to-face visit [17]. In SFHN, the population that receives an e-consult is similar to its overall racially/ethnically diverse patient population.

#### Effectiveness

Population Health. Timely access to specialty care is the main measure that has been used to evaluate the effectiveness of each electronic consultation system on population health. Two systems have reported a mean e-consult response time of 1–2 business days; the other two systems report that 91–95% of e-consults were completed within 3 business days. Wait times for in-person specialty care visits, measured by third next available specialty care in-person appointments, a widely-accepted metric of wait time for primary care access published by the Institute for Healthcare Improvement [18], declined in SFHN as a result of implementing the electronic consultation program [19]. These data are not available for the other systems. The percentage of e-consults associated with an avoidable in-person visit ranges from 37 to 82% in the delivery systems where e-consult is an adjunct to the traditional referral system and is approximately 23% in SFHN, where all requests for non-urgent ambulatory specialty care are initiated as e-consults [5, 20–22].

None of the systems in this case study have reported data on patient level outcomes, although providers consistently perceive e-consults as delivering high quality specialty care for patients. In prior survey studies, over 90% of PCPs participating in the BASE and Boston VA electronic consultation systems believed that e-consults provided faster specialty care for their patients and nearly three-quarters of Mayo clinic and SFHN PCPs agreed that e-consults provide “good medical care” [6, 8, 23, 24]. Additional data from each system demonstrate that 50–60% of specialists agree that e-consults provide good medical care [8, 24, 25].

All four healthcare delivery systems have documented positive and negative unintended consequences related to their electronic consultation implementation. In the BASE and SFHN systems, approximately 85–90% of PCPs self-reported high levels of educational value inherent to e-consult communication [26]. Additionally, 3.4% of e-consults in the BASE system resulted in initiation of a face-to-face referral when not originally contemplated by the PCP, a potentially important safety indicator [27]. Potential patient harms associated with e-consults have been evaluated at the Mayo clinic, in two specialties at SFHN, and in 5 specialties in the VA. A chart review of 187 e-consults with recommendations from the most common e-consult specialties at the Mayo clinic demonstrated that specialist e-consult recommendations were not completed 10% of the time [20]. In SFHN, among patients who received an e-consult and were not scheduled for an in-person general surgery or gastroenterology face-to-face appointment, 1–2% of patients experienced emergency department visits or hospital stays related to the content of their e-consult [28]. However, none of these harms were clearly due to lapses in communication during the consultation process. In one VA medical center, only 0.8% of 61,931 e-consults to 5 medical specialties did not have a specialist response. Chart review demonstrated that 6.3% of e-consults did not have appropriate specialist follow-up after the initial communication and 7.4% did not have appropriate documented PCP follow-up [22].

Patient satisfaction. Patient satisfaction with e-consults has been evaluated in three of the four systems with surveys and focus groups and has been generally positive. Nearly one-half of patients who received an initial in-person endocrinology consultation reported being open to the idea of receiving an e-consult from the BASE system instead, considering it a viable alternative to many but not all face-to-face appointments for diabetes and endocrine conditions [9]. In SFHN, focus group participants also appreciated improved access to specialty care with e-consult but voiced a desire to receive more information about the PCP-specialist communication [29]. A group of 15 Veterans from the Pittsburg VA who completed a survey about e-consults reported a mean satisfaction score of 5/5 on a Likert scale [30].

Care Team Satisfaction. Published survey data from BASE and VA systems suggest > 80% of PCPs and > 50% of specialists are satisfied with the electronic consultation system [23, 24]. Unpublished data from SFHN and Mayo Clinic are similar. Interviews with PCPs and specialists from three systems corroborate these data [7, 31, 32].

Financial Implications. Only two of the four systems in this case series have available cost data; both have suggested cost savings attributed to decreased face-to-face specialty care appointments and decreased travel costs incurred by patients. Taking into account the technological start-up costs of electronic consultation implementation, administrative and personnel costs associated with system maintenance, specialist remuneration based on time-spent per e-consult communication, and intentionally avoided specialist in-person visits based on PCP report, the BASE system calculated an average cost of $16.71 CAD per e-consult and a break-even point for the system with a total volume of 7818 e-consults [11]. A national VA study examining 217,000 e-consults to 13 different specialties between 2011 and 2013 suggested $2,800,000 savings from avoided patient travel to in-person specialty care visits. The VA evaluation did not take into account any fixed costs associated with implementation of the electronic consultation system [17].

#### Adoption

All four systems are multi-specialty, with over 50 participating medical, surgical, pediatric, women’s health, and mental health specialties. In 2016, the percentage of PCPs that requested e-consults for their patients using the BASE, Mayo and SFHN systems ranged from 75 to 100%, with a greater percentage in the system that mandated the use of e-consults for initial ambulatory specialty care requests. Such data are not readily available from the VA, due to its decentralized implementation. In all U.S, systems, participating PCPs included family physicians, internal medicine physicians, nurse practitioners and physician assistants; pediatricians also used the Mayo and SFHN services [26, 32, 33]. Only family physicians and nurse practitioners submitted e-consults in the BASE system [34]. Demographic characteristics and level of experience of PCPs who submit e-consults versus those who do not are not available.

#### Implementation

Poor access to specialty care and existing inefficiencies in the traditional referral process were the common main drivers for implementation across all health systems [23, 24, 35, 36]. Despite this overarching similarity, local reinforcing factors and challenges influenced implementation [36–38]. For example, the types of e-consult specialties offered by each system were influenced by requests from local PCPs as well as the presence/absence of local specialty physician champions. Local strategies to ensure success that were similar across all 4 systems included primary care workflow re-design and active involvement of physician champions. Customizable templates and integration with the EHR were local reinforcing factors that were unique to the VA. Implementation barriers common to all four sites included increased and/or different PCP and specialist workload, lack of clinical oversight, and concern about the legal implications of electronic communication.

#### Maintenance

Each of these four electronic consultation systems started as pilot projects with few participating specialties and referring providers. Submitting e-consults is now routine practice in each system. Common reinforcing factors for growth have included: investment in a core implementation team with a program manager, health information technology expert, PCP champion, and specialist champion; development of fair reimbursement strategies for providers; and a commitment to system quality improvement [35]. In the VA, widespread growth has been attributed to de-centralized activities. Decisions about workflow management and template development have been delegated to local specialty services, allowing hundreds of individual services differing in size, staffing, wait times, and clinical settings, to tailor the e-consult program according to local needs and constraints [32]. This contrasts with the BASE and SFHN system, where e-consult activities have been centrally managed.

## Discussion

Electronic consultation is a disruptive innovation that has challenged the status quo of specialty health care delivery traditionally limited to face-to-face patient-provider encounters [19, 32]. Disruptive innovations often provide the most value to individuals or groups unable to participate in successful status quo processes [39]. It is not surprising that early adopters of electronic consultation systems, including some in this case series, have been motivated by challenges in access to, or efficiency of, specialty care delivery through traditional face-to-face consults alone. As this analysis demonstrates, while the organizational drivers of implementation were similar across our early adopter health systems, unique system-level facilitators and barriers influenced local adoption and implementation, ultimately affecting the final design of each system. Despite these differences, implementation of this disruptive innovation was consistently associated with improved patient access to timely, perceived high-quality specialty expertise and few negative consequences.

Disruptive innovation theory suggests that widespread dissemination of electronic consultation programs will occur only when their positive impact is clearly obvious and the quality of care delivery is acceptable to all populations, including those that were perhaps less vulnerable at the outset. Further dissemination of electronic consultation will thus depend on the collection and publication of data to comprehensively demonstrate its value and to close existing gaps in knowledge related to Reach, Effectiveness and Adoption. In one of their landmark reports, the Institute of Medicine stated that standardized metrics for health and health care could provide benchmarks for progress and system performance [40]. With that guiding principle, we propose a set of metrics for electronic consultation rooted in the Quadruple Aim that stem directly from the implementation analysis depicted in this manuscript (Table 3). These metrics have been vetted by diverse stakeholders in electronic consultation, including leaders in healthcare delivery, payers, and professionals with expertise in quality, and provide an important step forward to promote high-value dissemination. Next steps will need to include validation on a national level through a formal consensus-building process.

**Table 3:** Proposed core effectiveness metrics for electronic consultation programs, using the Quadruple Aim framework

Arm of the Quadruple Aim	Measure	Definition	Rationale
Financial	E-consult management	Percentage of e-consults that are not scheduled for a face-to-face visit in the ensuring 12 months/Total number of e-consults per year	Calculate the number of avoidable face-to-face visits
Financial	Out of network specialty care requests	Number of out-of-network specialty care requests/Total number of specialty care requests	Examine changes in out-of-network specialty care visits
Population Health	Time to third next available new in-person appointment for e-consult specialties	Third next available new patient appointment if patient calls to make appointment	Direct measure of impact on specialty care access
Population Health	Demographics of patients who received an e-consult compared to in-person specialty care	Insurance status of patients who received at least one e-consult/Insurance status of all patients who received specialty expertise (e-consult + in-person specialty visits)	Program reach
			Impact on equity
Population Health	PCP capacity	Percentage of PCPs who self-report educational value of the e-consult program on a survey	Effectiveness of e-consult
Population Health	Number of specialties offering e-consult and what they are	Raw number of specialties offering e-consult	Measure of adoption
Population Health	Unclosed loop by PCP	Number of specialist responses that are not read by PCP per year/Total number of specialist responses via e-consult per year	Patient safety; unanticipated impact
Population Health	Unclosed loop by Specialist	Number of e-consults that did not receive a specialist response per year/total number of e-consults per year	Patient safety; unanticipated impact
Population Health	Average time to e-consult response	Average lapsed number of days between time e-consult was generated and time specialist responded	Access to specialty care
Care team experience	PCP satisfaction/dissatisfaction	Percentage of PCPs who report satisfaction with the program on a survey	Program sustainability
Care team experience	Specialist satisfaction/dissatisfaction	Percentage of specialists who report satisfaction with the program	Program sustainability
Care team experience	Medical Assistant/Nurse/Referral Coordinator satisfaction/dissatisfaction	Percentage of non-MD team primary care team members who report satisfaction with the program	Program sustainability
Patient experience	Satisfaction with access to specialty care in general	Percentage of patients who report satisfaction with access to specialty care pre- and post- implementation.	Program sustainability
Patient experience	Concerns about limitations in care	N/A	
Patient experience	Patient acceptability of having an e-consult	N/A	
Patient experience	Travel/time saved by patients for avoided clinic visits	Number of hours that patients must forgo for each in-person visit	Business case for managed care plans

By applying the RE-AIM framework for program implementation, we were able to identify characteristics associated with greater *Reach* and *Adoption* of electronic consultation programs. Consistent with data from newer programs, local penetration across patient and PCP populations was highest in the delivery system with an integrated electronic consultation and referral system, in which an e-consult was the initial step in the referral pathway [41]. Overall reach was lower in systems with separate electronic consultation and electronic referral systems, though PCP adoption in those systems was higher when a priority was placed on primary care system re-design. By contrast, *Effectiveness* measures were similar amongst the systems. E-consult response time was excellent in all 4 sites, as was the perceived quality of care inherent to electronic consultation, the educational value to referring providers, and overall program satisfaction by diverse stakeholders. Importantly, these findings from early U.S. and Canadian adopters are consistent with newer U.S. programs [42–45]. Analyses of European e-consult systems have also reported similar data: levels of provider satisfaction and educational benefit exceeding 90% [46, 47] and response time for humanitarian e-consults for international providers who are geographically isolated from specialists have been reported to be 1–3 days [48].

The use of validated frameworks for comparative program evaluation facilitates the identification of knowledge gaps. From this analysis, we note a paucity of data about which patient populations are directly benefiting from e-consults. Demographic data for patients who receive an e-consult compared to the general population served by a healthcare delivery system would be helpful for stakeholders concerned about replicating health inequities associated with prior technology-driven programs [49]. Additionally, while electronic consultation systems have improved timely access to specialty expertise, data associating improved clinical outcomes and electronic consultations are lacking. One lone trial has demonstrated decreased emergency department utilization among patients randomized to receive a cardiology e-consult compared to those randomized to participate in the usual cardiology referral process [3]. Since the clinical benefits of in-person specialty care visits have not been robustly demonstrated for all specialties, the lack of a gold standard to which e-consults can be compared may be contributing to the paucity of data and is one reason why we have not included clinical benefits in our list of proposed core evaluation metrics. Nevertheless, this area represents a large gap in the *Effectiveness* literature and may warrant a large, multi-specialty clinical trial. Similarly, the financial implications and return on investment for electronic consultation implementation need to be further examined.

This study also demonstrates that *Implementation* and *Maintenance* could be facilitated by policies that provide guidance on the legal implications of electronic consultation, ways to provide clinical oversight, and ideal remuneration strategies to account for increased provider workload while remaining aligned with the strategic directions of ongoing health care reform. The California Medicaid 2020 waiver [50] is a promising policy example that promotes the use of e-consult across California, as it provides financial incentives for specialty care re-design among public delivery systems, including funds for non-traditional specialty care encounters.

Despite providing important data about the implementation of electronic consultation programs across diverse healthcare delivery systems, the analysis is limited by the small number of programs included in this case series. Also, this study does not prove a direct relationship between electronic consultation program implementation and patient outcomes, such as specialty care access. Nonetheless, the comparable magnitude of impact across the four programs pertinent to Reach and Effectiveness is reassuring, as are similar data that have emerged from newer programs.

## Conclusion

Successful implementation of electronic consultation programs is occurring across diverse health care delivery systems in North America, consistently improving timely access to specialist expertise. The use of an evaluation framework with common metrics, as proposed here, is important to promote ongoing adoption and diffusion. As additional reach, outcome, safety, and cost data emerge from existing and nascent programs, electronic consultation may become a standard mode of specialty care delivery.

## Additional file

Additional file 1: **Table S1.** RE-AIM dimensions, definitions and evaluation questions pertinent to electronic consultation implementation. Description: The 5 RE-AIM domains are listed, with definitions of each domain, and example metrics or questions for each domain pertinent to the implementation of electronic consultation. (DOCX 31 kb)

## Abbreviations

BASE: Building Access to Specialists through eConsultation
EHR: Electronic health record
LIHN: Local Health Integration Network
PCP: Primary care provider
RE-AIM: Reach, Effectiveness. Adoption, Implementation, Maintenance
SFHN: San Francisco Health Network
VA: Veterans Affairs
VAMC: Veterans Affairs Medical Centers
ZSFG: Zuckerberg San Francisco General Hospital
