# Supplementary material for: Evaluating diverse electronic consultation programs with a common framework
Source: BMC Health Serv Res. 2018 Oct 24;18:814. doi: 10.1186/s12913-018-3626-4 (PMC6201558; doi:10.1186/s12913-018-3626-4)
Supplement: Supplementary file 1 — Table S1. RE-AIM dimensions, definitions and evaluation questions pertinent to electronic consultation implementation. Description: The 5 RE-AIM domains are listed, with definitions of each domain, and example metrics or questions for each domain pertinent to the implementation of electronic consultation. (DOCX 31 kb) [file 12913_2018_3626_MOESM1_ESM.docx]

| Supplementary Exhibit. RE-AIM dimensions, definitions and evaluation questions pertinent to electronic consultation implementation. | | | |
| --- | --- | --- | --- |
| **RE-AIM Dimension** | **Definition** | | **Questions pertinent to e-consult** |
| **Reach** (individual level) | The absolute number, proportion, and representativeness of individuals who are willing to participate in a given initiative, intervention, or program. | | What percentage of the population received an e-consult? |
|  |  |  | Were patients who received an e-consult representative of the overall practice setting? |
|  |  |  | Did the program reach those in most need? |
| **Effectiveness** (individual level) | The impact of an intervention on important outcomes including potential negative effects, as depicted in the domains of the **Quadruple Aim**. | **Population Health** | Did e-consults result in more timely specialty access? |
|  |  |  | How did the e-consult program impact quality of care? |
|  |  |  | What were some unintended adverse consequences? |
|  |  | **Patient Experience** | How did it impact patient experience? |
|  |  | **Care team experience** | What was provider satisfaction with the program? |
|  |  | **Financial implications** | What did the program cost as implemented? |
| **Adoption**  (setting and/or organizational level) | The absolute number, proportion, and representativeness of settings and intervention agents (people who deliver the program) who are willing to initiate a program. | | What percentages of eligible PCPs used the system? |
|  |  |  | Were participating PCPs representative of others in the health care delivery system? |
|  |  |  | What specialties were represented in the e-consult system? |
| **Implementation** (setting and/or organizational level) | Fidelity of implementation and adaptions made during delivery. | | What barriers to implementation were identified and how were they addressed? |
|  |  |  | What predisposing factors (drivers) affected implementation? |
|  |  |  | What enabling factors were required to support the implementation? |
| **Maintenance** (individual and setting/organizational levels) | The extent to which a program or policy becomes institutionalized or part of the routine organizational practices and policies. | | How did the program evolve and grow? |
|  |  |  | What reinforcing factors were required to maintain the e-consult service? |
